# Novel applications of Machine Learning in cheminformatics

**DOI:** 10.1186/s13321-018-0301-z

**Published:** 2018-09-06

**Authors:** Ola Spjuth

**Affiliations:** Department of Pharmaceutical Biosciences, Uppsala University, Box 591, 751 24 Uppsala, Sweden

In recent years we have seen a renewed interest in Artificial Intelligence and Machine Learning in cheminformatics, and the idea of collecting, structuring and making use of Big Data in e.g. drug discovery has become a popular topic [1, 2]. Deep Learning methods are also making their way into cheminformatics and drug discovery [3, 4], further contributing to the increased attention. Data sets relevant for Machine Learning in cheminformatics are increasing in numbers and size, for example the ChEMBL database has grown from 2.4 million activity values in 2010 (ChEMBL version 02) to over 14 million activity values in 2017 (ChEMBL version 23) [5]. This has been propelled by the trend of organizations and companies depositing data sets in ChEMBL for public use.

An important topic of Machine Learning is quantifying the uncertainty of the predictions produced by classification and regression models. Conformal Prediction is a methodology where predictors provide information about their own accuracy and reliability [6]. In contrast to traditional Machine Learning that delivers point estimates, Conformal Prediction yields a prediction region that contains the true value with probability equal to or higher than a predefined level of confidence. Such a prediction region can be obtained under the assumption that the observed data is exchangeable. Conformal Prediction has been demonstrated in cheminformatics [7], with the attractive property that it offers a compelling alternative to the topic of applicability domain determination [8]. Using Conformal Prediction, the size of the prediction region will be larger if the compound is ‘non-conforming’ to the training set.

This article collection in Journal of Cheminformatics features three articles on the topic of applications of Conformal Prediction and deep learning.

Larger datasets and demanding methods such as Deep Learning necessitates high-performance e-infrastructures. Ahmed et al. [9] present an iterative Conformal Prediction approach for virtual screening implemented in Apache Spark on cloud computing resources, and show how the number of docked compounds can be reduced significantly with a Machine Learning augmented approach compared to traditional dock-all strategies. Svensson et al. [10] uses Conformal Prediction to predict what strategy generates the highest gain in a high-throughput screening setting. The authors show that by learning from a subset of the compound library, inferences on what compounds to screen next can be made by predictive models, resulting in more efficient screening. De la Vega de León et al. [11] provide insights into how missing data affect multitask prediction methods, using Deep Learning and Bayesian probabilistic matrix factorization.

This collection in Journal of Cheminformatics includes a set of extended versions of the top ranking papers presented in the 6th Symposium on Conformal and Probabilistic Prediction with Applications (COPA 2017) at Karolinska Institutet, Stockholm, Sweden on June 14–16, 2017. Further, the collection was open for contribution from other authors. All papers went through a regular reviewing process and were properly revised, if necessary, prior to acceptance.
